# Protocol for minimized-bias profiling of liver and visceral adipose tissue in mice using integrated single-nucleus RNA sequencing

**DOI:** 10.1016/j.xpro.2026.104699

**Published:** 2026-07-08

**Authors:** Lei Li, Jonathan G. Pol, Christophe Desterke, Wanchao Hu, Nicolas Trainel, Morgane Benardeau, Manuela Lizarralde-Guerrero, Maria Chiara Maiuri, Guido Kroemer, Gabriel Perlemuter, Anne-Marie Cassard, Cosmin Sebastian Voican

**Affiliations:** 1 Université Paris-Saclay, INSERM UMR 996-MI2, Microbiome and Metabolome in Liver Disease: from Susceptibility to Treatment, 91400 Orsay, France; 2 Centre de Recherche des Cordeliers, Equipe Labellisée Par La Ligue Contre le Cancer, Université de Paris Cité, Sorbonne Université, INSERM U1138, Institut Universitaire de France, 75006 Paris, France; 3 INSERM UMRS 1310, Université Paris-Saclay, Faculté de Médecine, 94270 Le Kremlin-Bicêtre, France; 4 Université Paris-Saclay, Faculté de Médecine, 94270 Kremlin-Bicêtre, France; 5 Department of Molecular Medicine and Medical Biotechnologies, University of Naples Federico II, 80131 Naples, Italy; 6 Université Paris-Saclay, INSERM US23 / CNRS UAR 3655, Metabolomics and Cell Biology Platforms, Institut Gustave Roussy, 94800 Villejuif, France; 7 Institut Du Cancer Paris CARPEM, Department of Biology, Hôpital Européen Georges Pompidou, AP-HP, 75015 Paris, France; 8 Service Hépato-gastroentérologie et Nutrition, Hôpital Antoine-Béclère, AP-HP Université Paris-Saclay, 92140 Clamart, France

**Keywords:** Single Cell, Sequencing, RNAseq, Metabolism, Molecular Biology

## Abstract

Single-nucleus RNA sequencing (snRNA-seq) enables transcriptomic profiling of tissues that are difficult to dissociate. Here, we present an integrated protocol combining tissue-specific nuclei extraction and selective enrichment to isolate high-quality nuclei from fresh mouse liver and visceral adipose tissue (VAT). We describe steps for single-nucleus extraction, magnetic enrichment, BD Rhapsody capture, cDNA synthesis, whole-transcriptome amplification, index PCR, library quality control, and shallow sequencing-based assessment of cell-type recovery, while separating whole-liver and non-parenchymal cell workflows to reduce dissociation bias.

## Highlights

• Isolates nuclei from fresh MASLD mouse liver and VAT for snRNA-seq
• Separates whole-liver and NPC workflows to reduce dissociation bias
• Defines tissue-specific enrichment for liver, VAT, and NPC-derived nuclei
• Provides recovery benchmarks and QC guidance for BD Rhapsody workflows

## Abstract (editor highlights)

Publisher’s note: Undertaking any experimental protocol requires adherence to local institutional guidelines for laboratory safety and ethics.

## Graphical abstract

**Figure:**
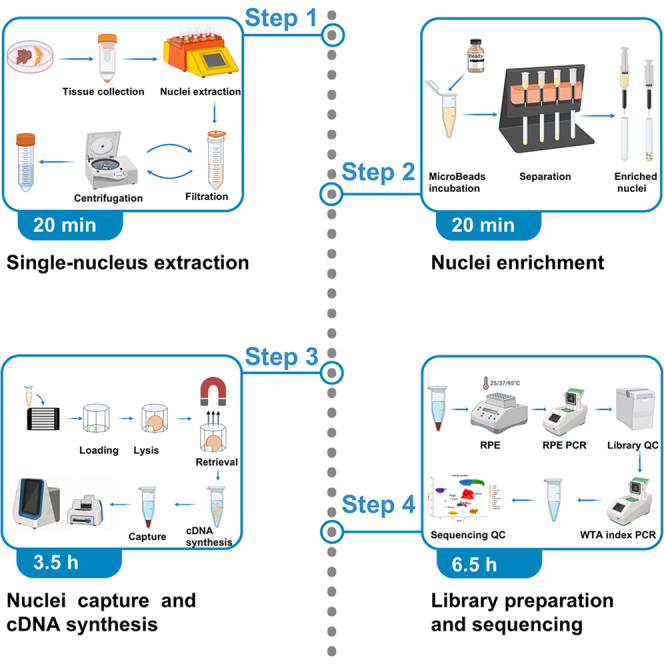

## Before you begin

SnRNA-seq enables transcriptional profiling of tissues in which obtaining intact cell suspensions is challenging, such as frozen tissue samples.^1^^,^^2^ In the context of MASLD, hepatocytes and adipocytes present unique technical challenges: they are large, fragile, metabolically sensitive cells that can be substantially damaged during conventional tissue dissociation, introducing significant bias in downstream analyses.^3^ In particular, steatotic hepatocytes and lipid-laden adipocytes typically exceed the effective size range supported by many microwell- or microfluidic-based single-cell platforms.^4^^,^^5^ Under these conditions, only smaller and likely less metabolically representative cells are captured for single-cell transcriptomics, which can lead to profound selection bias and loss of insight into the physiology of the major parenchymal cells.^5^

This protocol provides a detailed, step-by-step workflow for isolating high-quality nuclei from hepatocytes and NPCs in livers of MASLD mice and can be readily extended to VAT. Distinct isolation strategies are applied according to tissue and cell type. For hepatocyte-rich liver tissue and VAT, nuclei are directly extracted from freshly dissected tissue using the Miltenyi Biotec Nuclei Extraction Kit, with optional enrichment to improve nuclei purity. In contrast, NPCs are first isolated at the cellular level prior to nuclei extraction. Enrichment is not recommended for NPC-derived nuclei, as it can substantially reduce nuclei recovery and yield, necessitating a tissue-specific enrichment strategy. Although this protocol was optimized for MASLD mouse tissues, it is readily adaptable to other fragile, lipid-rich, or heterogeneous tissue samples.

All buffers, kits, and equipment required for nuclei isolation and BD Rhapsody™ processing should be prepared and pre-cooled in advance, as detailed in the materials and equipment section, before starting the experiment.

### Innovation

The main innovation of this protocol lies in its tissue-adapted workflow design. It integrates Miltenyi Biotec nuclei extraction and magnetic enrichment with the BD Rhapsody™ HT platform to provide a complete workflow from fresh MASLD mouse tissues to single-nucleus capture, WTA library preparation, and shallow sequencing-based quality control.

This workflow addresses two practical limitations of applying snRNA-seq to MASLD tissues. First, hepatocytes and adipocytes are large, fragile, and lipid-rich, which makes conventional whole-cell dissociation prone to cell loss and selection bias. Second, direct whole-liver nuclei preparation can underrepresent non-parenchymal populations because hepatocyte-derived nuclei predominate. To address this, whole liver tissue and pre-isolated NPC fractions are processed separately before downstream single-nucleus analysis.

A further refinement of this protocol is the tissue-specific use of magnetic enrichment. Together with recovery benchmarks and downstream library and sequencing quality control (QC), these adaptations define a practical approach for technically challenging, lipid-rich metabolic tissues. Specifically: (i) tissue handling and dissociation conditions were adapted for fragile, lipid-rich liver and VAT, including use of pre-cooled gentleMACS™ Octo Coolers and calibrated RNase inhibitor concentrations not specified in the standard manufacturer protocol, (ii) whole liver tissue and pre-isolated NPC fractions are processed separately to reduce hepatocyte dominance and improve representation of non-parenchymal populations, (iii) magnetic enrichment was empirically evaluated across tissue sources and found to improve microscopic cleanliness for liver- and VAT-derived nuclei, whereas it caused marked nuclei loss in NPC-derived samples and is therefore not recommended for this fraction, and (iv) a post-elution 30 μm filtration step was added after magnetic separation to remove aggregates that accumulate during enrichment, which is not part of the standard Anti-Nucleus MicroBeads protocol.

### Institutional permissions

Male wild-type C57BL/6J mice (Janvier Laboratories, Le Genest, France), 8 weeks old at diet initiation and euthanized after 26 weeks of western diet feeding, were housed in humidity- and temperature-controlled rooms on a 12-h light-dark cycle. An enriched environment was provided to minimize anxiety, agonistic behaviors, and stress. All procedures were performed in accordance with national and institutional guidelines for the use of animals in research. The present study was conducted under the procedure (#47097-2024012516265646 v4) approved by the local Ethics Committee.

### Preparation of nuclei extraction and enrichment

**Timing: 10 min**

***Note:*** Pre-cool a centrifuge, buffers, and consumables to 4°C before beginning. Perform all nuclei extraction steps in a cold room (4°C) whenever possible to preserve nuclear integrity. The reagent volumes below are optimized for processing a single sample; scale proportionally for multiple samples.

1. Prepare the nuclei extraction buffer supplemented with RNase inhibitor.
  a. In a 50 mL Falcon tube, add 5 mL of pre-chilled Nuclei Extraction Buffer with RNase inhibitor to a final concentration of 0.2 U/μL.
  b. Mix thoroughly by gentle inversion (do not vortex).
  c. Transfer 2 mL of the supplemented buffer into a pre-cooled gentleMACS™ C Tube and keep on ice until use.
2. Prepare nuclei resuspension buffer as described in Materials and equipment.

**CRITICAL:** Degas all buffers before use to prevent bubble formation during the subsequent magnetic enrichment step.

***Note:*** It is highly recommended to maintain nuclei concentration below 5 × 10^6^/mL throughout preparation to minimize nuclei aggregation. Excessively high nuclei concentrations may lead to increased aggregation, reduced capture efficiency, and potential clogging or doublet formation during downstream processing. If the concentration exceeds this threshold, dilute the nuclei suspension with appropriate ice-cold resuspension buffer before proceeding.

***Note:*** For tissues with high endogenous RNase activity, consider increasing the RNase inhibitor concentration in both nuclei extraction and resuspension buffers to 0.4 U/μL to prevent RNA degradation.

### Preparation of nuclei capture and cDNA synthesis

**Timing: 10 min**

***Note:*** The preparation described here can proceed concurrently with Step 17 (Anti-Nucleus MicroBeads incubation) to optimize workflow efficiency.

3. Store Vybrant® DyeCycle™ Green Stain at 15°C–25°C protected from light.
4. Thaw all reagents (except enzymes) from the BD Rhapsody™ cDNA Kit at 15°C–25°C, then place on ice. Store enzymes at −25°C to −15°C until use.
5. Prepare Sample Buffer-RI (Sample Buffer with RNase inhibitor).
  a. Pipette 5 mL of Sample Buffer into a tube and add RNase inhibitor to achieve a final concentration of 0.2 U/μL.
  b. Use this Sample Buffer-RI throughout all BD Rhapsody™ cartridge workflow steps prior to the lysis step.
6. Place all required reagents on ice: Sample Buffer-RI, 1 M DTT, Lysis Buffer, BD Rhapsody™ Enhanced Cell Capture Beads, and Bead Wash Buffer.
7. Incubate Cartridge Wash Buffers 1 and 2 at 15°C–25°C for 30 min before use.
8. Verify that a SmartBlock™ Thermoblock (1.5 mL) or equivalent is installed on the thermomixer and set to 37°C according to the experimental protocol.
9. Set an additional thermomixer to 80°C and allow it to preheat.

**CRITICAL:** Keep the isolated nuclei suspended in Sample Buffer-RI and maintain them on ice or at 4°C throughout the procedure. Use ice-cold buffers for all steps and minimize pipetting to prevent mechanical damage to nuclear membranes.

### Preparation of library construction

**Timing: 5 min**

10. Program one heat block to 95°C and set two thermomixers to 37°C and 25 °C, respectively.
11. In a 15-mL conical tube, prepare 10 mL of freshly made 80% (v/v) ethyl alcohol by mixing 8 mL of absolute ethanol with 2 mL of nuclease-free water. Vortex for approximately 10 seconds to ensure complete mixing.
12. Thaw all reagents (except enzymes) from the BD Rhapsody™ WTA Amplification Kit at 15°C–25°C. Immediately transfer thawed reagents to ice.

***Note:*** Prepare fresh 80% ethanol and use it within 24 h. Adjust the volume of 80% ethanol according to the number of libraries being processed.

## Key resources table

**Table:** 

REAGENT or RESOURCE	SOURCE	IDENTIFIER
**Biological samples**		

Male wild-type C57BL/6J mice, 8 weeks old at diet initiation; tissues collected after 26 weeks of western diet feeding	Janvier Labs	Ethics approval #47097-2024012516265646 v4

**Chemicals, peptides, and recombinant proteins**		

PBS	Gibco	Cat#10010023
DMEM	Gibco	Cat#10566016
Absolute ethanol	Major supplier	N/A
Nuclease-free water	Major supplier	N/A
DAPI Staining Solution	MILTENYI BIOTEC	Cat#130-111-570
RNase inhibitor, 40000 U/mL	New England Biolabs	Cat#M0314
BD Stain Buffer (FBS)	BD Biosciences	Cat#554656
Proteinase K, 800 U/mL	New England Biolabs	Cat#P8107S
Vybrant® DyeCycle™ Green Stain	Thermo Fisher	Cat#V35004

**Critical commercial assays**		

Nuclei Extraction Buffer	MILTENYI BIOTEC	Cat#130-128-024
Liver Dissociation Kit, mouse	MILTENYI BIOTEC	Cat#130-105-807
Anti-Nucleus MicroBeads	MILTENYI BIOTEC	Cat#130-132-997
BD Rhapsody™ cDNA Kit	BD Biosciences	Cat#633773
BD Rhapsody™ Enhanced Cartridge Reagent Kit	BD Biosciences	Cat#667052
BD Rhapsody™ 8-Lane Cartridge	BD Biosciences	Cat#666262
BD Rhapsody™ WTA Amplification Kit	BD Biosciences	Cat#633801
Qubit™ dsDNA HS Assay Kit	Thermo Fisher	Cat#Q32851
Agilent High Sensitivity DNA Kit	Agilent	Cat#5067-4626
MACS BSA Stock Solution	MILTENYI BIOTEC	Cat#130-091-376
BD® OMICS-One Dual Index Kit	BD Biosciences	Cat#571899
Agencourt® AMPure® XP magnetic beads	Beckman Coulter	Cat#A63880

**Other**		

LS Columns	MILTENYI BIOTEC	Cat#130-042-401
MACS SmartStrainers (30 μm)	MILTENYI BIOTEC	Cat#130-098-458
MACS SmartStrainers (70 μm)	MILTENYI BIOTEC	Cat#130-098-462
MACS SmartStrainers (100 μm)	MILTENYI BIOTEC	Cat#130-098-463
GentleMACS C Tubes	MILTENYI BIOTEC	Cat#130-093-237
Eppendorf® LoBind® Tubes	BD Biosciences	Cat#570751
Hemocytometer	NANOENTEK	Cat#DHC-N01
Hemocytometer adapter	BD Biosciences	Cat#633703
Centrifuge and rotor with adapters for 5-mL Falcon® tubes and 15-mL tubes.	Major supplier	N/A
GentleMACS Octo Dissociator with Heaters	MILTENYI BIOTEC	Cat#130-096-427
MidiMACS™ Separator	MILTENYI BIOTEC	Cat#130-042-302
MACS MultiStand	MILTENYI BIOTEC	Cat#130-042-303
GentleMACS™ Octo Coolers	MILTENYI BIOTEC	Cat#130-130-533
60 mL reagent reservoir self-standing	BD Biosciences	Cat#666626
Reagent reservoir (10 mL)	VistaLab	Cat#3054-1012
Reagent reservoir (25 mL)	VistaLab	Cat#3054-1002
Falcon® tube with nuclei strainer cap	Corning	Cat#352235
Corning® 96-well polypropylene cluster tube	Corning	Cat#4413
DNA LoBind® tubes, 1.5-mL	Eppendorf	Cat#0030108051
DNA LoBind® tubes, 2.0-mL	Eppendorf	Cat#022431048
Low-retention, filtered pipette tips (20 μL, 200 μL, 1000 μL)	Major supplier	N/A
Deep 96-Well 2 mL Polypropylene plate	BRAND	Cat#BR701354-24EA
Pre-moistened cleaning wipes	Major supplier	N/A
Lint-free wipes	Major supplier	N/A
BD Rhapsody Scanner	BD Biosciences	Cat#633701
BD Rhapsody HT Xpress System	BD Biosciences	Cat#700036499
BD Rhapsody™ P8xP1200 μL Pipette-HTX	BD Biosciences	Cat#500066280
Eppendorf ThermoMixer C	Eppendorf	Cat#5382000023
SmartBlock Thermoblock 1.5-mL	Eppendorf	Cat#5360000038
Incubator	Major supplier	N/A
Pipettes (P10, P20, P200, P1000)	Major supplier	N/A
Multi-channel pipette (P200)	Major supplier	N/A
Vortex Mixer	Major supplier	N/A
Digital timer	Major supplier	N/A
6-Tube magnetic separation rack	New England Biolabs	Cat#S1506S
0.2-mL PCR 8-strip tubes	Major supplier	N/A
Axygen® 96-Well PCR Microplates	Corning	Cat#PCR96HSC
Microcentrifuge for 1.5–2.0-mL tubes	Major supplier	N/A
Microcentrifuge for 0.2-mL tubes	Major supplier	N/A
Low-profile magnetic separation stand for 0.2 mL, 8-strip tubes	V&P Scientific, Inc.	Cat#VP772F4-1
Magnetic Stand-96	Thermo Fisher	Cat#AM10027
MicroAmp Clear Adhesive Film	Thermo Fisher	Cat#4306311
Agilent® 2100 Bioanalyzer	Agilent	Cat#G2940CAG
Qubit™ 3.0 Fluorometer	Thermo Fisher	Cat#Q33216
Qubit™ Assay Tubes	Thermo Fisher	Cat#Q32856
PCR thermal cycler	Major supplier	N/A
Heat block	Major supplier	N/A

## Materials and equipment

**Table:** Nuclei resuspension buffer

Reagent	Final concentration	Amount for 1 sample
PBS, pH 7.2	—	8.964 mL
MACS BSA Stock Solution, 10% BSA	0.04% BSA	36 μL
Nuclei Extraction Buffer	1:7 dilution	1.5 mL
RNase inhibitor, 40000 U/mL	0.2 U/μL	52.5 μL

## Step-by-step method details

### Extract nuclei from mouse liver, VAT, and isolated NPCs

**Timing: 20 min**

This section describes the rapid extraction of intact nuclei from fresh mouse liver tissue, isolated NPC fractions, and VAT using Miltenyi’s Nuclei Extraction Buffer and gentleMACS™ Octo Dissociator. Perform all steps on ice or at 4°C unless otherwise specified.

***Note:*** This protocol is adapted from the manufacturer’s instructions with carefully validated modifications to optimize nuclei recovery specifically from liver, NPC fractions, and adipose tissue.

***Note:*** Fresh tissues are recommended for optimal nuclei recovery and integrity. Frozen tissues may be processed when fresh material is not available, but nuclei quality should be assessed microscopically before proceeding to enrichment or single-nucleus capture. For automated tissue dissociation and nuclei isolation using gentleMACS™ Octo Dissociator, place the gentleMACS™ C Tubes in pre-cooled gentleMACS™ Octo Coolers (stored at −20 °C) to ensure optimal performance. After the nuclei suspension is obtained, handle samples gently during all pipetting steps to preserve nuclear integrity.

1. At the time of sacrifice, anesthetize mice to achieve rapid and deep anesthesia using Zoletil (tiletamine/zolazepam, 500 mg/kg) in combination with Rompun (xylazine, 20 mg/kg).
  a. Perform cardiac blood collection to clear circulating blood from the liver.
  b. Immediately harvest up to 200 mg of fresh liver tissue or visceral adipose tissue (VAT).
  c. Rinse the tissue in ice-cold (4 °C) DMEM to remove residual blood.
  d. Mince the tissue into small fragments (approximately 1–2 mm) using sterile scissors.
  e. Promptly transfer the tissue fragments to a pre-cooled gentleMACS™ C Tube containing 2 mL of nuclei extraction buffer supplemented with RNase inhibitor.

***Note:*** For frozen tissue samples, cut specimens into pieces smaller than 50 mg before freezing in liquid nitrogen and store at −80 °C. When processing frozen material: (i) avoid repeated freeze-thaw cycles, which accelerate RNA degradation and nuclear fragmentation, (ii) transfer frozen tissue pieces directly into pre-chilled nuclei extraction buffer without allowing thawing prior to mechanical dissociation, and (iii) assess nuclei integrity microscopically by DAPI staining (Step 10) before proceeding to enrichment or single-nucleus capture. Note that frozen tissue is not compatible with the upstream viable NPC isolation step; for NPC profiling, only fresh tissue should be used. For additional guidance on expected differences in nuclei quality when processing frozen versus fresh material, see Troubleshooting, Problem 6.

***Note:*** For previously isolated NPCs, remove the supernatant after centrifugation and proceed with nuclei extraction by adding 2 mL of nuclei extraction buffer directly to the cell pellet and transferring the mixture into a pre-cooled gentleMACS™ C Tube. In this protocol, NPC fractions are obtained from fresh liver tissue using the Miltenyi Biotec Liver Dissociation Kit (Cat#130-105-807), which provides a standardized approach to enrich non-parenchymal cell populations prior to nuclei isolation. No additional purification step is required before nuclei extraction. Alternative NPC isolation methods may also be compatible, but variations in isolation strategy may influence the relative composition of recovered cell populations. We therefore recommend using a consistent NPC isolation method to ensure reproducible nuclei quality and cell-type composition.

2. Close the gentleMACS™ C Tube and place it on the gentleMACS™ Octo Dissociator. Fit the Coolers onto the C Tube and run the “4C_nuclei_1” program.
3. After the program completes, detach the C Tube and place it immediately on ice.

***Note:*** A tissue-dependent 5–10 min on-ice incubation can enhance tissue lysis; however, avoid extended incubation (>15 min) to prevent over-lysis and loss of nuclear integrity.

4. Apply the nuclei suspension to a pre-cooled MACS™ SmartStrainer (70 μm pore size) placed on a 15 mL low-binding collection tube.

***Note:*** The use of low-binding tubes is recommended at this stage and throughout subsequent steps to minimize nuclei loss due to surface adhesion. Pre-wetting of the SmartStrainer is not required under standard conditions, but may be used as a troubleshooting measure if substantial nuclei loss is observed during filtration (see Troubleshooting, Problem 3).

5. Wash the SmartStrainer with 2 mL of ice-cold nuclei extraction buffer. Rinse the used C Tube with 1 mL ice-cold nuclei extraction buffer and add the rinse to the SmartStrainer.
6. Remove the SmartStrainer from the collection tube and centrifuge the nuclei suspension at 300 × *g* for 5 min at 4°C. Carefully aspirate and completely discard the supernatant without disturbing the nuclei pellet at the tube bottom.
7. Resuspend the nuclei pellet in 2 mL of ice-cold resuspension buffer by gently pipetting up and down 10 times.
8. Pass the nuclei suspension through a MACS™ SmartStrainer (30 μm) placed on a 15 mL collection tube to remove remaining cell debris and aggregates.
9. Discard the SmartStrainer and collect the nuclei suspension. Gently mix by pipetting to ensure uniform distribution.
10. Add DAPI Staining Solution to a small aliquot (50–100 μL) of the nuclei suspension to achieve a final concentration of 0.25 μg/mL. Mix gently but thoroughly by pipetting (avoid vigorous mixing).
11. Incubate the stained nuclei for 5 min on ice protected from light.
12. Load the stained nuclei suspension onto a hemocytometer slide.
13. Count the nuclei according to the hemocytometer manufacturer’s instructions.

***Note:*** Use at least four counting chambers to ensure accurate counting. Keep the remaining unstained nuclei suspension on ice for subsequent steps.

### Enrichment of isolated nuclei using Anti-Nucleus MicroBeads

**Timing: 20 min**

Enrich isolated nuclei using Anti-Nucleus MicroBeads and magnetic separation according to the manufacturer’s instructions. Keep nuclei on ice throughout the procedure and proceed immediately to single-nucleus capture.

**Figure 1:**
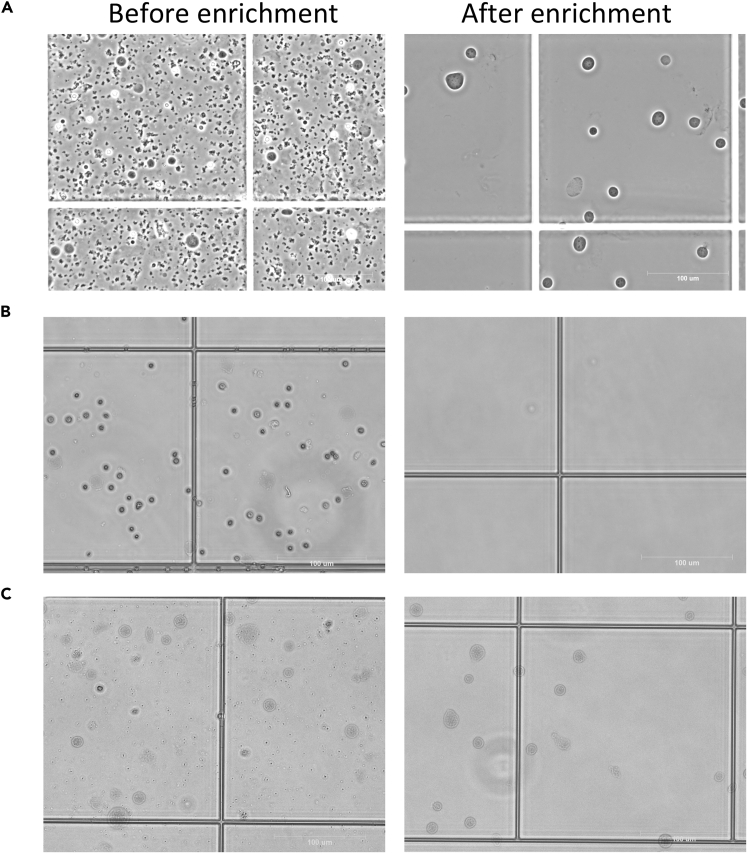
Bright-field assessment of nuclei before and after magnetic enrichment Representative bright-field images of nuclei isolated from mouse liver, non-parenchymal cell (NPC), and visceral adipose tissue (VAT) samples before and after magnetic enrichment. (A) Liver-derived nuclei. (B) NPC-derived nuclei. (C) VAT-derived nuclei. Scale bar, 100 μm.

***Note:*** This enrichment workflow is based on Miltenyi’s Anti-Nucleus MicroBeads manual protocol with optimized modifications to accommodate tissue-dependent differences in nuclei composition (Figure 1).

***Note:*** The enrichment step yields tissue-dependent results. Select the appropriate sub-step below based on your tissue source (hepatocytes, NPCs, or VAT).

***Note:*** Throughout all manipulations of the nuclei suspensions, employ gentle pipetting techniques to minimize hydrodynamic shear stress, which could damage nuclear membranes.

***Note:*** Observation-Nuclei directly extracted from hepatocyte-rich liver tissue (Figure 1A) or VAT (Figure 1C) show improved microscopic cleanliness and reduced visible debris following magnetic enrichment, as assessed by DAPI staining and microscopic inspection. In contrast, applying the same enrichment procedure to nuclei derived from NPC fractions leads to a marked reduction in nuclei recovery, with very few nuclei remaining after enrichment (Figure 1B). NPC-derived nuclei showed acceptable microscopic cleanliness before enrichment, whereas magnetic enrichment caused marked nuclei loss without clear improvement in sample quality (Figure 1B). Based on these observations, we do not recommend applying magnetic enrichment to NPC-derived nuclei. The underlying cause of this differential response is not fully defined but may relate to differences in nuclei size, composition, or compatibility with the Anti-Nucleus MicroBeads system. Therefore, for other tissue types or cell sources, we recommend performing a pilot test to determine whether enrichment improves nuclei quality and recovery.

14. After counting, collect 1 × 10^6^ nuclei in a new tube for enrichment. Centrifuge the suspension at 300 × *g* for 5 min at 4°C. Carefully aspirate and discard the supernatant.

***Note:*** Enrichment is not recommended for NPC-derived nuclei; these samples should proceed directly to Step 27 (Staining nuclei with DyeCycle™ Green for scanner-based counting).

***Note:*** All enrichment steps are performed in 15 mL low-binding tubes unless otherwise specified.

15. Resuspend the nuclei pellet in 450 μL of nuclei separation buffer.
16. Add 50 μL of Anti-Nucleus MicroBeads to the nuclei suspension.
17. Mix thoroughly by gentle inversion and incubate for 15 min at 4°C.

***Note:*** During this incubation, prepare reagents for the “Single-nucleus capture and cDNA synthesis” section.

18. After incubation, add 2 mL of nuclei separation buffer and mix gently. Immediately proceed to magnetic separation.

***Note:*** Keep this volume constant regardless of sample input.

19. Position a pre-cooled MACS Column into the magnetic field of a suitable MACS Separator.
20. Equilibrate the column by passing 3 mL of nuclei separation buffer through it.
21. Apply the nuclei suspension to the center of the column reservoir. Collect the flow-through containing debris and unlabeled nuclei.
22. Wash the column twice by passing 1 mL of nuclei separation buffer through it each time. Combine all wash fractions with the initial flow-through.
23. Remove the column from the magnetic separator and place it onto a pre-cooled 5 mL flow cytometry tube kept on ice.
24. Add 2 mL of nuclei separation buffer to the column and immediately elute magnetically labeled nuclei by firmly pushing the plunger into the column reservoir.
25. Keep the nuclei suspension on ice until proceeding to the next section.

### Single-nucleus capture and cDNA synthesis

**Timing: 3.5 h**

In this section, nuclei are loaded into the BD Rhapsody™ 8-Lane Cartridge for high-throughput single-nucleus capture using the BD Rhapsody™ HT Single-Cell Analysis System according to the manufacturer’s workflow.

***Note:*** This protocol section follows the BD Rhapsody™ HT System User Guide and incorporates validated modifications optimized specifically for nuclei capture and downstream processing from MASLD tissues.

***Note:*** Handle nuclei suspensions with care to minimize mechanical stress; avoid vigorous mixing or excessive pipetting that may compromise nuclear membrane integrity.

***Note:*** Always use low-retention filtered pipette tips and LoBind tubes. Change pipette tips between each pipetting step to prevent cross-contamination and bubble formation caused by residual liquid in the pipette tip.

***Note:*** Prepare nuclei as close as possible to the loading step to maintain optimal sample quality and viability.

***Note:*** Keep all reagents on ice, unless otherwise specified.

***Note:*** Inspect the Lysis Buffer visually for any precipitate before use. If precipitate is observed, incubate at 15 °C–25°C for 1 h until completely clear. Mix gently by inversion (do not vortex). Once clear, return the buffer to ice and maintain at 4°C until use.

***Note:*** Retain the cartridge foil pouch and desiccant for storage of partially used cartridge. Place the waste collection container and Cluster Tube in the BD Rhapsody™ HT Xpress System drawer. Carefully remove the seal from the cartridge inlet according to the number of lanes being used. For correct instrument configuration, set the Front Slider to Waste and the Retrieval Slider to Inactive (Back) position.

***Note:*** Priming the cartridge with ethyl alcohol followed by an air purge ensures complete wetting of the microwell array during the Prime/Wash procedure (Step 26b). Small random bubbles (<3 mm diameter) during priming do not affect cartridge performance. If large bubbles (>3 mm) appear in any lane, use Prime/Wash mode to aspirate and dispense air, then repeat Step 26a with 100% ethyl alcohol. Perform this procedure only during priming steps. Uneven fluid flow or asymmetric liquid fronts between lanes do not impact cartridge performance. Small residual volumes in pipette tips after dispensing are normal; discard tips appropriately.

***Note:*** Use a P20 pipette to remove any buffer pooling at the cartridge inlet, aspirating at an angle to prevent unintentional uptake of buffer from the microwell array. Perform this step only during cartridge priming.

26. Cartridge Priming and Wash Procedure.
  a. Load 50 μL of 100% ethyl alcohol into each cartridge lane using the EtOH Prime mode on the BD Rhapsody™ P8×1200 μL digital pipette.
  b. Load 380 μL of air in Prime/Wash mode.
  c. Add 380 μL of Cartridge Wash Buffer 1 in Prime/Wash mode and incubate for 1 min at 15 °C–25 °C.
  d. Load 380 μL of air in Prime/Wash mode.
  e. Add a second 380 μL volume of Cartridge Wash Buffer 1 in Prime/Wash mode and incubate for 3 min at 15 °C–25 °C.
  f. Load 380 μL of air in Prime/Wash mode.
  g. Finally, add 380 μL of Cartridge Wash Buffer 2 in Prime/Wash mode and incubate for up to 4 h at 15 °C–25 °C.
27. Staining nuclei with DyeCycle™ Green for scanner-based counting.
  a. Centrifuge the nuclei suspension from Step 25 at 400 × g for 5 min at 4 °C.
    i. Carefully aspirate the supernatant.
    ii. Leave approximately 20 μL of residual volume.
    iii. Add cold Sample Buffer-RI to reach a final volume of 620 μL.
  b. Add 2.6 μL of 5 mM DyeCycle™ Green to the 620 μL nuclei suspension in cold Sample Buffer-RI (approximately 1:240 dilution).
  c. Gently mix by pipetting to ensure homogeneous staining.
  d. Keep the stained nuclei suspension on ice for 5 min, protected from light.
  e. Filter the suspension through a pre-cooled 30 μm SmartStrainer mounted on a 2-mL LoBind tube.
  f. Immediately count the nuclei using the BD Rhapsody™ scanner.
    i. Before counting, gently pipet-mix to ensure nuclei are evenly suspended.
    ii. Load 10 μL of the suspension into an INCYTO™ disposable hemocytometer.
28. Counting and preparing single-nucleus suspensions for cartridge loading.
  a. Insert the hemocytometer into the Hemocytometer Adapter, then tap Scan.
  b. Place the adapter onto the scanner tray and tap Continue.
  c. Select “Hemocytometer” as the protocol, then enter or select the experiment name, sample name, and user.
  d. Tap Side A or Side B, then select “Start Side A Scan” or “Start Side B Scan” (Nuclei Count).
  e. Once the scan is complete, tap OK.
  f. To scan the other side, tap Scan again, enter a new sample name, and repeat Steps 28d–28e. Alternatively, tap Eject to remove the adapter, then tap Done.
  g. Tap Analysis and select the experiment name to review total nuclei concentration and viability.
  h. Proceed as follows:
    i. If the nuclei concentration is ≤1,000 nuclei/μL, proceed to Step 28i.
    ii. If the nuclei concentration is >1,000 nuclei/μL, dilute the nuclei suspension in cold Sample Buffer-RI to ∼200–800 nuclei/μL. Repeat Steps 28a–28g, and then Step 28i.
      ***Note:*** Under standard conditions, starting from ∼1 × 10^6^ nuclei, the final nuclei concentration after enrichment and processing typically remains above 500 nuclei/μL. If the nuclei concentration falls below ∼100 nuclei/μL after filtration (Step 27e), first check for potential sample loss during enrichment or centrifugation. If the low yield reflects limited starting material rather than technical loss, the sample can still be loaded onto the BD Rhapsody™ system, but reduced capture efficiency and lower final nuclei numbers should be expected.
  i. Tap Prepare to display the Samples Calculator screen.
  j. Dispose of the hemocytometer properly. Minimize the time between nuclei samples counting and pooling and single-nucleus loading into the cartridge.
  k. Use the Samples Calculator to determine the required stock nuclei and buffer volumes for preparing a 380 μL nuclei suspension for loading.
  l. Select the appropriate cartridge type (for the BD Rhapsody™ 8-Lane Cartridge, choose 0120) and plan to load up to 100,000 nuclei per lane.
  m. Prepare the nuclei suspension according to the calculated volumes displayed on the scanner. Ensure each stock sample is well suspended by gentle pipet-mixing before pooling.
  n. Transfer each prepared single-nucleus suspension to a pre-cooled 96-deep-well plate kept on ice for multi-lane loading.
29. Loading nuclei into the BD Rhapsody™ Cartridge.
  ***Note:*** Gently mix the nuclei suspension with a multi-channel pipette to ensure complete resuspension before loading. Set the pipette to Load mode and proceed immediately with the loading process; small air bubbles at the inlet or outlet of the cartridge, outside of the microwell array, do not affect cartridge performance.
  a. Load 380 μL of air into one lane using the Prime/Wash pipette mode, then immediately load 320 μL of the nuclei suspension into the designated lane using the Load mode.
    ***Note:*** If condensation is visible on the cartridge surface, gently wipe it off with a lint-free tissue to ensure clear imaging during scanning.
  b. Incubate the cartridge at 15 °C–25°C for 8 min. If the cartridge is placed directly on the scanner, set an 8-minute delay before tapping “Start Cell Load Scan” (Nuclei Load step) to allow incubation.
  c. While the cartridge is incubating, prepare the BD Rhapsody™ Enhanced Cell Capture Beads according to the manufacturer’s instructions (see Step 30).
  d. Image the nuclei in the cartridge by running the scanner step “Cell Load”.
  e. When scanning is complete, tap OK, then Eject the cartridge, remove it from the scanner and verify that image analysis has successfully started.
  f. Place the cartridge into the BD Rhapsody™ HT Xpress System and set the BD Rhapsody™ P8×1200 μL Pipette to Prime/Wash mode.
  g. Per lane, sequentially load 380 μL of air, then 380 μL of cold Sample Buffer-RI, another 380 μL of air, and a final 380 μL of cold Sample Buffer-RI in Prime/Wash mode.
30. Preparing BD Rhapsody™ Enhanced Cell Capture Beads.
  ***Note:*** Keep BD Rhapsody™ Enhanced Cell Capture Beads on ice before and during preparation. To maximize bead recovery, never vortex bead suspensions; instead, mix gently by pipetting. Use low-retention filtered tips and LoBind tubes, and maintain all bead suspensions at low temperature.
  a. Place the BD Rhapsody™ Enhanced Cell Capture Beads tube on a magnetic rack for 1 min to collect beads, then carefully remove and discard the storage buffer.
  b. Remove the tube from the magnet and add 380 μL of cold Sample Buffer-RI.
  c. Gently resuspend the beads by pipetting and place the tube on ice.
  d. Transfer each bead suspension to a pre-cooled 96-deep-well plate for multi-lane loading and keep the plate on ice until the Nuclei Load scan is complete.
  e. Once the Nuclei Load scan and analysis have started, proceed to bead loading (Step 31).
31. Loading and Washing BD Rhapsody™ Enhanced Cell Capture Beads.
  a. Place the cartridge into the BD Rhapsody™ HT Xpress System.
  b. Using the BD Rhapsody™ P8×P1200 μL Pipette, first dispense 380 μL of air per lane in Prime/Wash mode to prepare the lanes for bead loading.
  c. Set the pipette to Mix mode and gently resuspend the capture Beads until the beads are completely resuspended. Ensure the tips reach the well bottom to avoid air bubbles, then discard the tips.
  d. With a fresh set of tips, switch the pipette to Load mode, confirming that no air bubbles are present in the tips before loading.
  e. Immediately dispense 320 μL of Enhanced Cell Capture Beads into each lane of the cartridge.
  f. Incubate the cartridge at 15 °C–25°C for 3 min to allow beads to settle and distribute evenly.
  g. On the scanner interface, run the “Bead Agitation” step.
  h. After agitation, tap OK, then Eject to remove the cartridge from the scanner and confirm that bead analysis has started.
  i. Return the cartridge to the BD Rhapsody™ HT Xpress System. Prepare Sample Buffer-RI in a 10 mL reagent reservoir according to the number of lanes to be processed using the table provided.

**Table:** 

Component	1 lane	2 lanes	3 lanes	4 lanes	5 lanes	6 lanes	7 lanes	8 lanes
Sample Buffer-RI (mL)	0.38	2.00	2.80	3.60	4.30	5.10	5.90	6.60
  j. Set the BD Rhapsody™ pipette to Prime/Wash mode.
  k. Sequentially load each lane with 380 μL of air, 380 μL of cold Sample Buffer-RI, a second 380 μL of air, and a final 380 μL of cold Sample Buffer-RI, all in Prime/Wash mode.
  l. Perform the scanner step “Bead Wash”.
  m. Once the scan is complete, tap OK and then select Eject to remove the cartridge from the scanner. Confirm that the analysis has started running after the scan.
    ***Note:*** When nuclei are processed using the BD Rhapsody™ System, the reported viability will be 100%, as all nuclei are positive for DyeCycle™ Green. This value does not represent the actual viability of the loaded nuclei; however, the cell counts generated by the system remain accurate. Accordingly, cartridge metrics obtained from the BD Rhapsody Scanner should be interpreted with this limitation in mind.
32. Lysing nuclei.
  ***Note:*** When opening the DTT tube, hold it vertically to prevent loss of the inert, non-oxygen-containing gas overlaying the solution. Do not tilt the tube, as this can cause the inert gas to escape. If fewer than four or eight lanes are processed, immediately reseal the DTT tube and store it at −20 °C. Use Lysis Buffer with DTT within 24 h, then discard any remaining solution.
  a. Prepare Lysis buffer supplemented with DTT by adding 75.0 μL of 1 M DTT to one 15-mL bottle of Lysis Buffer and verifying that the DTT addition box is checked.
  b. Vortex the mixture for 5–10 s, then aliquot the Lysis Buffer with DTT into a 10-mL or 25-mL reagent reservoir according to the number of lanes, using the table below. This lysis mix will be used in Step 33.

**Table:** 

Component	1 lane	2 lanes	3 lanes	4 lanes	5 lanes	6 lanes	7 lanes	8 lanes
Lysis Buffer (mL)	1	3.75	5.60	7.50	9.40	11.25	13.10	15.00
  c. Transfer 1 mL of the prepared Lysis Buffer with DTT to a new Eppendorf Tube® and add 50 μL of Proteinase K. Mix thoroughly and keep on ice.
    ***Note:*** Prepare sufficient Lysis Buffer-DTT-Proteinase K mixture according to the number of lanes (280 μL per lane) and transfer the required volume to a pre-cooled 96-deep-well plate for multi-lane loading.
  d. Place the cartridge into the BD Rhapsody™ HT Xpress System and set the BD Rhapsody™ P8×P1200 μL Pipette to Lysis mode.
  e. Load 280 μL of Lysis Buffer containing DTT and Proteinase K into each lane in Lysis mode.
  f. Incubate the cartridge at 15°C–25°C for 5 min.
  g. Maintain the recommended lysis duration to ensure optimal nuclei recovery while preserving RNA integrity.
    ***Note:*** Before bead retrieval, remove any excess buffer pooled at the cartridge inlet with a P20 pipette, aspirating at an angle to avoid accidentally drawing buffer from the microwell array.
33. Retrieving BD Rhapsody™ Enhanced Cell Capture Beads.
  a. Place the Cluster Tube (8-tube strip) into the BD Rhapsody™ HT Xpress System drawer, labeling each tube appropriately.
  b. Set the front slider to BEADS, and move the top Retrieval slider into the ACTIVE position so that the Retrieval magnet contacts the BD Rhapsody™ 8-Lane Cartridge. Keep it active for 1 min to collect beads.
  c. Set the BD Rhapsody™ P8×P1200 μL Pipette (or P1200 μL Pipette) to Retrieval mode, and aspirate 1,000 μL of Lysis Buffer with DTT.
  d. Before the 1-min activation period ends, firmly seal the pipette against the cartridge gasket.
    i. When the minute has elapsed, switch the Retrieval magnet to INACTIVE.
    ii. Immediately dispense the buffer into the cartridge.
    iii. Discard the used tip.
  e. Move the front slider to OPEN, remove the Cluster Tube with adapter, and place it on ice. Then switch the front slider to WASTE, but keep the waste container for later use.
  f. Place a new Cluster Tube (8-tube strip) into the BD Rhapsody™ HT Xpress System drawer and label each tube appropriately. Repeat Steps 33b–33e.
    ***Note:*** Repeat the bead retrieval steps to collect as many beads as possible.
  g. Wipe any residual liquid from the cartridge outlet with a lint-free tissue, and perform the scanner step Retrieval.
  h. After the scan, tap OK, then Eject and Done to confirm analysis has started.
  i. Keep the partially used cartridge on a flat surface while proceeding to Washing BD Rhapsody™ Enhanced Cell Capture Beads and Reverse Transcription.
34. Washing BD Rhapsody™ Enhanced Cell Capture Beads.
  a. Detach the Cluster Tubes from the adapter. Gently mix the beads obtained from the two retrieval rounds of the same lane by pipetting, and transfer the combined beads into a 2-mL LoBind tube. Keep the tube on ice.
  b. If beads remain in the Cluster Tube, rinse with 100 μL of Lysis Buffer containing DTT, and transfer the rinse to the same LoBind tube.
  c. Place the tube on a 1.5-mL magnetic rack for up to 2 min, then carefully remove the supernatant. Avoid leaving residual buffer or air bubbles, as the presence of Lysis Buffer may interfere with the reverse transcription reaction.
  d. Remove the tube from the magnet and add 1.0 mL of cold Bead Wash Buffer. Mix gently by pipetting.
    ***Note:*** When processing multiple samples, keep tubes on ice between washing steps.
  e. Return the tube to the magnet for ≤2 min and discard the supernatant.
  f. Repeat the wash once more with 1.0 mL of cold Bead Wash Buffer. Mix gently, place on ice, and proceed to reverse transcription within 30 min after washing.
35. Performing Reverse Transcription.
  a. Ensure that the SmartBlock™ Thermoblock for ThermoMixer® C is pre-set to 37 °C.
  b. In a pre-amplification workspace, prepare the cDNA mix in a new 1.5-mL or 2.0-mL LoBind tube placed on ice by adding the following reagents:

**Table:** 

Component	For 1 library (μL)	For 1 library + 20% overage (μL)	For 4 libraries + 20% overage (μL)	For 8 libraries + 20% overage (μL)
RT Buffer	40.0	48.0	192.0	384.0
dNTP	20.0	24.0	96.0	192.0
RT 0.1 M DTT	10.0	12.0	48.0	96.0
Bead RT/PCR Enhancer	12.0	14.4	57.6	115.2
RNase Inhibitor	10.0	12.0	48.0	96.0
Reverse Transcriptase	10.0	12.0	48.0	96.0
Nuclease-Free Water	98.0	117.6	470.4	940.8
**Total**	**200.0**	**240.0**	**960.0**	**1,920.0**
    ***Note:*** A 20% overage is included in all reaction mixes to compensate for pipetting variability and reagent loss during handling. This does not indicate preparation of fractional libraries, as the relative proportions of all components remain unchanged.
  c. Mix the reaction for 5–10 s by vortexing, then spin down for 5–10 s and place the tube back on ice.
  d. Place the tube containing washed BD Rhapsody™ Enhanced Cell Capture Beads on a 1.5-mL magnetic rack for ≤ 2 min. Remove and discard the supernatant.
  e. Add 200 μL of the prepared cDNA mix to the beads and mix gently by pipetting. Keep the bead suspension on ice until it is transferred in the next step.
  f. Transfer the bead suspension to a new 1.5-mL LoBind tube.
  g. Incubate the bead suspension on the SmartBlock™ at 1,200 rpm and 37°C for 20 min.
    ***Note:*** Gentle shaking is critical for efficient reverse transcription.
  h. During the incubation, review the image analysis to verify that the quality metrics have been met.
  i. After incubation, place the tube on ice.
36. Treating Samples with Exonuclease I.
  a. Set one thermomixer to 37°C and another to 80 °C.
    ***Note:*** Avoid using a heat block for Exonuclease I inactivation, as temperatures above 80°C may compromise bead-associated oligonucleotides.
  b. In a pre-amplification workspace, prepare the Exonuclease I mix in a new 1.5-mL or 2.0-mL LoBind tube on ice by combining the following reagents:

**Table:** 

Component	For 1 library (μL)	For 1 library + 20% overage (μL)	For 4 libraries + 20% overage (μL)	For 8 libraries + 20% overage (μL)
10× Exonuclease I Buffer	20.0	24.0	96.0	192.0
Exonuclease I	10.0	12.0	48.0	96.0
Nuclease-Free Water	170.0	204.0	816.0	1,632.0
**Total**	**200.0**	**240.0**	**960.0**	**1,920.0**
  c. Mix by vortexing for 5–10 s, spin down for 5–10 s, and place the tube back on ice.
  d. Position the tube containing BD Rhapsody™ Enhanced Cell Capture Beads with cDNA mix on a 1.5-mL magnetic rack for ≤ 2 min. Remove and discard the supernatant.
  e. Remove the tube from the magnet and add 200 μL of the prepared Exonuclease I mix. Mix gently by pipetting.
  f. Incubate the tube on the thermomixer at 1,200 rpm and 37°C for 30 min.
  g. Without shaking, incubate the bead suspension at 80°C for 20 min to inactivate the enzyme.
    ***Note:*** Do not exceed the specified temperature or incubation time.
  h. Cool the tube on ice for approximately 1 min, then spin down for 5–10 s. Remove and discard the supernatant.
  i. Add 200 μL of cold Bead Resuspension Buffer to the tube and mix gently by pipetting.
    **Pause point**: The Exonuclease I-treated beads can be stored at 2–8°C for up to 12 months. These beads are subsequently used for the library preparation step.
37. Washing Used Lanes and Storing the BD Rhapsody™ 8-Lane Cartridge.
  a. After completing the experiment, set the front slider of the BD Rhapsody™ HT Xpress System to WASTE.
  b. Prepare nuclease-free water and 100% ethyl alcohol in a 10-mL reagent reservoir, adjusting the total volume according to the number of lanes used.

**Table:** 

Component	1 lane (mL)	2 lanes (mL)	3 lanes (mL)	4 lanes (mL)	5 lanes (mL)	6 lanes (mL)	7 lanes (mL)	8 lanes (mL)
Nuclease-free water	0.38	2.00	2.00	2.50	2.50	3.00	3.50	4.00
100% ethyl alcohol	0.05	2.00	2.00	2.00	2.00	2.00	2.00	2.00
  c. Rinse each used lane sequentially using the BD Rhapsody™ P8×P1200 μL Pipette.
    i. Dispense 380 μL of air in Prime/Wash mode.
    ii. Dispense 380 μL of nuclease-free water in Prime/Wash mode and incubate for 1 min at 15 °C–25 °C.
    iii. Dispense 380 μL of air in Prime/Wash mode.
    iv. Dispense 50 μL of 100% ethyl alcohol in EtOH Prime mode.
    v. Dispense 380 μL of air in Prime/Wash mode.
  d. After washing, use a lint-free wipe to remove any residual liquid from the cartridge surface. Ensure that the seals of the unused lanes remain intact.
  e. Store the cartridge flat in its original pouch with desiccant, sealed in a double zipper bag, and keep it at 15 °C–25°C in the dark.
  f. Clean the BD Rhapsody™ HT Xpress System with 10% bleach or 70% ethyl alcohol, and properly discard the waste container and used buffers.

### mRNA whole-transcriptome analysis

**Timing:** 6.5 h

This section describes the preparation of single-nucleus whole-transcriptome mRNA libraries after nuclei capture on the BD RhapsodyTM HT Single-Cell Analysis System for sequencing on compatible sequencing platforms.

***Note:*** This library preparation workflow is based on the BD Rhapsody™ System mRNA WTA Library Preparation Protocol and includes slight modifications to accommodate nuclei-derived material.

***Note:*** When working with BD Rhapsody™ Enhanced Cell Capture Beads, use low-retention filtered tips and LoBind® tubes. Bring Agencourt® AMPure® XP magnetic beads to 15 °C–25°C before use.

**CRITICAL:** Never vortex the BD Rhapsody™ Enhanced Cell Capture beads. Pipet-mix only.

38. Random priming and extension (RPE).
  a. In a new 1.5-mL LoBind® tube, pipet the following reagents, mix the prepared random primer mix gently by pipetting and keep it at 15 °C–25 °C.

**Table:** 

Component	**For 1 library (μL)**	1 library with 20% overage (μL)	4 libraries with 20% overage (μL)	8 libraries with 20% overage (μL)
WTA extension buffer	20.0	24.0	96.0	192.0
WTA extension primers	20.0	24.0	96.0	192.0
Nuclease-free water	134.0	160.8	643.2	1286.4
**Total**	**174.0**	**208.8**	**835.2**	**1670.4**
  b. Spin down the tube containing Exonuclease I-treated BD Rhapsody™ Enhanced Cell Capture Beads for 5–10 s, then place it on a 1.5-mL magnetic rack for ≤ 2 min until the supernatant clears. Discard the supernatant.
  c. Remove the tube from the magnet and resuspend the beads in 75 μL of Elution Buffer by pipet-mixing 10 times.
  d. Heat the suspension at 95°C for 5 min (no shaking), spin down for 5–10 s, and return it to the magnet for ≤ 2 min to remove the supernatant.
  e. Add 200 μL of Elution Buffer to the beads.
    i. Pipet-mix 10 times to resuspend the beads.
    ii. Keep the tube on ice if processing multiple libraries.
    iii. Spin down for 5–10 s.
    iv. Place the tube on the magnet for ≤2 min.
    v. Remove the supernatant completely.
  f. Add 87 μL of Random Primer Mix to the beads and pipet-mix 10 times to ensure uniform suspension. Keep any remaining primer mix at 15 °C–25°C for the second RPE reaction.
  g. Sequentially incubate the tube as follows:
    i. 95°C for 5 min (no shaking).
    ii. 37°C for 5 min at 1,200 rpm.
    iii. 25°C for 5 min at 1,200 rpm
      ***Note:*** If processing multiple libraries, keep the tubes on ice until all samples are denatured.
  h. Spin down for 5–10 s and keep the tube at 15 °C–25 °C.
  i. In a new 1.5-mL LoBind® tube, pipet the following Extension enzyme mix:

**Table:** 

Component	For 1 library (μL)	1 library with 20% overage (μL)	4 libraries with 20% overage (μL)	8 libraries with 20% overage (μL)
dNTP	8.0	9.6	38.4	76.8
Bead RT/PCR enhancer	12.0	14.4	57.6	115.2
WTA extension enzyme	6.0	7.2	28.8	57.6
**Total**	**26.0**	**31.2**	**124.8**	**249.6**
  j. Add 13 μL of Extension Enzyme Mix to the beads (final 100 μL volume). Keep at 15 °C–25°C until ready, and store any remaining mix on ice.
  k. Place the tube containing the extension enzyme mix and the BD Rhapsody™ Enhanced Cell Capture Beads into the programmed thermomixer. Run the thermomixer using the following settings:
    i. 25°C for 10 min at 1,200 rpm.
    ii. 37°C for 15 min at 1,200 rpm.
    iii. 45°C for 10 min at 1,200 rpm.
    iv. 55°C for 10 min at 1,200 rpm.
  l. After completion, place the tube on the magnet for ≤2 min and discard the supernatant.
  m. Add 205 μL elution buffer to the beads.
    i. Pipet-mix to fully resuspend the beads.
    ii. Incubate at 95°C for 5 min without shaking.
    iii. Open the lid to release pressure.
    iv. Mix at 1,200 rpm for 10 s.
  n. Place the tube on a magnetic rack for ≤2 min until the supernatant clears. Transfer 200 μL of RPE-containing supernatant to a new LoBind® tube and keep on ice.
  o. Repeat Steps 38f–38n for a second RPE reaction.
    i. Magnetically separate the second RPE reaction.
    ii. Transfer 200 μL of RPE-containing supernatant from the second reaction to the tube containing the first RPE supernatant.
    iii. Combine the two 200 μL supernatants to obtain 400 μL of pooled RPE product.
    iv. Store the pooled RPE product on ice.
    v. Store the beads at −20 °C.
39. RPE cleanup.
  ***Note:*** Perform the purification steps in the pre-amplification workspace. Allow the Agencourt® AMPure® XP magnetic beads to equilibrate to 15 °C–25°C before use.
  a. Vortex the AMPure® XP magnetic beads vigorously for 1 min to ensure complete resuspension.
  b. If the RPE product volume is <400 μL, adjust to 400 μL with elution buffer. Add 720 μL of AMPure® XP beads to the tube containing the 400 μL RPE supernatant. Pipet-mix at least ten times and spin down for 5–10 s.
  c. Incubate the beads-sample mixture at 15 °C–25°C for 10 min.
  d. Place the tube on a 1.5-mL magnetic rack for ∼5 min or until the supernatant clears. Remove and discard the supernatant.
  e. Keeping the tube on the magnet, gently add 1 mL of freshly prepared 80% ethyl alcohol.
  f. Incubate for 30 s, then remove and discard the supernatant.
  g. Perform a second 80% ethyl alcohol wash as in Steps 39e−39f.
  h. While the tube remains on the magnet, carefully remove any remaining liquid using a P20 pipette. Spin down the tube for 5–10 s while still wet, return the tube to the magnet, and remove any collected ethanol.
  i. Air dry the beads at 15 °C–25°C for 15–20 min until they appear matte and no longer glossy.
    ***Note:*** Do not overdry; cracked beads indicate overdrying.
  j. Remove the tube from the magnet and resuspend the bead pellet in 40 μL of elution buffer. Pipet-mix at least ten times to fully resuspend.
  k. Incubate at 15 °C–25°C for 2 min, spin down for 5–10 s, and place on the magnet for ∼30 s or until the solution clears. Transfer ∼40 μL of eluate to a new PCR tube. This is the purified RPE product.
  l. Keep the purified RPE product on ice until proceeding to RPE PCR.
    **Pause point**: Store the RPE product either on ice or at 4°C and use it within 24 h.
40. RPE PCR.
  a. Prepare RPE PCR mix in a new 1.5-mL LoBind® tube according to the following table:

**Table:** 

Component	For 1 library (μL)	1 library **with 20% overage (μL)**	4 libraries **with 20% overage (μL)**	8 libraries **with 20% overage (μL)**
PCR master mix	60	72	288	576
Universal oligo	10	12	48	96
WTA amplification primer	10	12	48	96
**Total volume**	**80**	**96**	**384**	**768**
  b. Pipet-mix the RPE PCR mix thoroughly and keep the mixture on ice.
  c. Add 80 μL of the prepared RPE PCR mix to 40 μL of purified RPE product. Pipet-mix at least ten times to generate the RPE PCR reaction mix.
  d. Split the reaction mixture into two 0.2-mL PCR tubes, each containing 60 μL of RPE PCR reaction mix.
  e. Transfer the reaction to the post-amplification workspace and run the PCR using the following program:

**Table:** 

Step	Cycles	Temperature (°C)	Time
Hot start	1	95	3 min
Denaturation	Refer to the Note below	95	30 s
Annealing	–	60	1 min
Extension	–	72	1 min
Final extension	1	72	2 min
Hold	1	4	∞
    ***Note:*** The total number of PCR cycles depends on the number of nuclei in the RPE sample. Use 16 cycles for samples containing approximately 100 nuclei, 13 cycles for 1,000–9,999 nuclei, 12 cycles for 10,000 nuclei, 11 cycles for 20,000 nuclei, 10 cycles for 40,000 nuclei, and 9 cycles for 80,000–100,000 nuclei. In our workflow, for samples containing approximately 40,000–80,000 nuclei, 10 cycles produced satisfactory library yield profiles without obvious over-amplification. Further optimization may be required depending on sample type and input quality.
  f. After PCR, spin down the tubes for 5–10 s to collect contents at the bottom.
    **Pause point**: The PCR can be safely held for 12–16 h at 4 °C.
41. RPE PCR cleanup.
  a. Bring Agencourt® AMPure® XP magnetic beads to 15 °C–25 °C.
  b. Vortex the AMPure® XP magnetic beads at high speed for 1 min to ensure full resuspension.
  c. Spin down the two 60-μL RPE PCR tubes for 5–10 s, then combine them into a new 1.5-mL tube (final volume = 120 μL).
    If the combined volume is <120 μL, adjust with elution buffer.
  d. Add 120 μL of AMPure® XP beads to the 120 μL RPE PCR product. Pipet-mix at least ten times and spin down for 5–10 s.
    ***Note:*** Avoid beads adhering to the tube lid, residual beads may affect downstream performance.
  e. Incubate the mixture at 15 °C–25°C for 5 min.
  f. Place the tube on a magnetic rack for ∼5 min or until the supernatant clears. Remove and discard the supernatant.
  g. Keeping the tube on the magnet, gently add 500 μL of fresh 80% ethanol. Incubate for 30 s, then remove and discard the supernatant without disturbing the bead pellet.
  h. Repeat the ethanol wash once (Step 41g) for a total of two washes.
  i. Keep the tube on the magnet and use a small-volume pipette to remove any remaining liquid. Allow the beads to air dry at 15 °C–25°C for ∼5 min or until matte in appearance.
    ***Note:*** Do not overdry; cracked beads indicate overdrying and may reduce recovery.
  j. Remove the tube from the magnet, add 40 μL of elution buffer, and pipet-mix at least ten times to fully resuspend the beads.
  k. Incubate at 15 °C–25°C for 2 min, spin down for 5–10 s, and place the tube on the magnet for ∼30 s or until clear.
  l. Carefully transfer ∼40 μL of eluate to a new 1.5-mL LoBind® tube. The purified RPE PCR product is ready for Index PCR.
    **Pause point**: Purified RPE PCR libraries can be stored at −20°C for up to 6 months or 4°C for up to 6 weeks.
42. Quality Check of RPE PCR Product.
  a. Measure the concentration of the RPE PCR products using a Qubit™ Fluorometer with the Qubit™ dsDNA HS Assay.
  b. Evaluate library size distribution using either an Agilent 2100 Bioanalyzer with the High Sensitivity DNA Kit or an Agilent 4200 TapeStation with High Sensitivity D1000 or D5000 ScreenTape assays.
    ***Note:*** A successful library should display a broad peak from ∼200 to 2,000 bp.
  c. Use the 200–600 bp range to calculate the DNA concentration for Index PCR setup.
    ***Note:*** Fragments outside the 200–600 bp range are removed during the double-sided cleanup after Index PCR.
43. WTA index PCR.
  a. Dilute purified RPE PCR products with nuclease-free water until the concentration of the 200–600 bp peak is 2 nM.
    i. If the product concentration is <2 nM, use the sample without dilution.
    ii. Example: If the Bioanalyzer shows 6 nM for the 200–600 bp peak, dilute threefold with nuclease-free water to reach 2 nM.
  b. Prepare WTA index PCR mix in a new 1.5-mL LoBind® tube using the following recipe:

**Table:** 

Component	For 1 library (μL)	1 library **with 20% overage (μL)**	4 libraries **with 20% overage (μL)**	8 libraries **with 20% overage (μL)**
PCR master mix	25	30	120	240
Library forward primer	5	6	24	48
Library reverse primer 1–4∗	5	6	–	–
Nuclease-free water	5	6	24	48
Total	40	48	168	336
    ***Note:*** Use a unique reverse primer for each library when preparing multiple libraries.
  c. Pipet-mix the WTA index PCR mix thoroughly and keep on ice until use.
  d. In a new 0.2-mL PCR tube, combine the WTA index PCR mix with diluted RPE PCR products as follows:
    i. Single library: 40 μL of WTA index PCR mix + 10 μL of 2 nM RPE PCR product (total 50 μL).
    ii. Multiple libraries: For each library, combine 35 μL of WTA index PCR mix with 5 μL of the appropriate reverse primer and 10 μL of 2 nM RPE PCR product.
  e. Pipet-mix each reaction at least ten times to ensure homogeneity.
  f. Run the following PCR program:

**Table:** 

Step	Cycles	Temperature (°C)	Time
Hot start	1	95	3 min
Denaturation	Refer to the Note below	95	30 s
Annealing	–	60	30 s
Extension	–	72	30 s
Final extension	1	72	1 min
Hold	1	4	∞
    ***Note:*** Use 9 PCR cycles for RPE PCR products at 1–2 nM, and 8 cycles for products at 2 nM; additional cycles may be needed if the concentration is <1 nM.
  g. When PCR is finished, spin down the tubes for 5–10 s to collect contents at the bottom.
    **Pause point**: The PCR can be safely held for 12–16 h at 4 °C.
44. WTA Index PCR Cleanup.
  a. Vortex AMPure® XP magnetic beads at high speed for 1 min until completely resuspended.
  b. Add 60 μL nuclease-free water to the WTA Index PCR product (final 110 μL).
  c. Pipet exactly 100 μL of the diluted product into a new 0.2-mL PCR tube.
    **CRITICAL:** Accurate volume is critical for proper size selection.
  d. Add 60 μL AMPure® XP beads to the 100 μL sample. Pipet-mix ≥10 times and spin down for 5–10 s.
    ***Note:*** Avoid beads on the tube lid, as residual beads may reduce yield.
  e. Incubate at 15 °C–25°C for 5 min.
  f. Place the tube on a magnetic rack for ∼3 min or until the supernatant clears.
  g. Carefully transfer 160 μL of clear supernatant to a new 0.2-mL tube without disturbing the beads.
    ***Note:*** The beads retained after Step 44g contain long fragments and should be discarded.
  h. Add 15 μL AMPure® XP beads to the recovered supernatant.
    i. Pipet-mix ≥10 times.
    ii. Spin down for 5–10 s.
    iii. Incubate at 15 °C–25°C for 5 min.
  i. Place the tube on the magnet until clear (∼3 min). Remove and discard the supernatant.
  j. Wash the beads twice with fresh 80% ethanol.
    i. Keeping the tube on the magnet, add 200 μL fresh 80% ethanol.
    ii. Incubate for 30 s.
    iii. Remove and discard the ethanol.
    iv. Repeat Steps 44j(i)–44j(iii) once for a total of two ethanol washes.
  k. While on the magnet, use a small pipette to remove any remaining liquid.
  l. Air dry at 15 °C–25°C for ∼30 s until matte.
    ***Note:*** Do not overdry; cracked beads indicate overdrying and can lower recovery.
  m. Elute the purified WTA Index PCR library.
    i. Remove the tube from the magnet.
    ii. Add 30 μL elution buffer.
    iii. Pipet-mix ≥10 times to fully resuspend the beads.
    iv. Incubate for 2 min at 15 °C–25 °C.
    v. Spin down for 5–10 s.
    vi. Place the tube on the magnet until the solution clears (∼30 s).
  n. Transfer ∼30 μL eluate to a new 1.5-mL LoBind® tube. This is the final sequencing-ready WTA Index PCR library.
    **Pause point**: The purified libraries can be stored at −20°C for up to 6 months before sequencing.
45. Quality Check of WTA Index PCR Product.
  a. Measure the concentration of the Index PCR libraries using a Qubit™ Fluorometer with the Qubit™ dsDNA HS Assay.
  b. Evaluate library size distribution using either an Agilent 2100 Bioanalyzer with the High Sensitivity DNA Kit or an Agilent 4200 TapeStation with High Sensitivity D1000 or D5000 ScreenTape assays.

***Note:*** A Qubit™ reading above 1 ng/μL is expected. Electropherogram profiles should display a primary peak between approximately 250 and 1,000 bp. Example traces for liver, NPC, and VAT libraries are provided in Figure 2.

**Figure 2:**
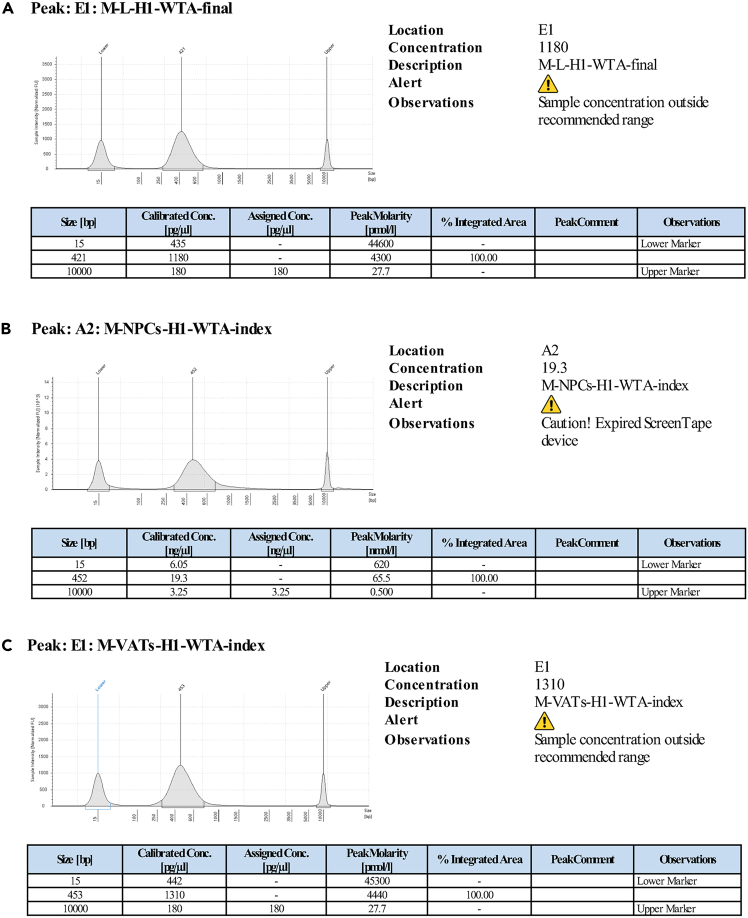
Electropherogram-based quality control of WTA index PCR libraries Representative electropherograms of WTA index PCR products generated from nuclei isolated from (A) liver, (B) non-parenchymal cell (NPC) fractions, and (C) visceral adipose tissue (VAT). Library size distribution and concentration were assessed using a microfluidic capillary electrophoresis system. All libraries show the expected fragment size range with lower and upper markers and were subsequently used for sequencing.

## Expected outcomes

This protocol enables rapid and reproducible isolation of nuclei from fresh mouse liver and VAT, followed by single-nucleus capture, cDNA synthesis, and WTA library preparation using the BD Rhapsody™ HT platform. By combining tissue-specific nuclei extraction, selective enrichment, and separate processing of liver tissue and NPC fractions, the workflow is designed to reduce dissociation-associated bias and improve recovery of nuclei from fragile, lipid-rich, and heterogeneous metabolic tissues.

Prior to enrichment, nuclei extraction yield should be verified by manual hemocytometer counting with DAPI staining (Steps 10–13); expected yields vary depending on tissue mass, quality, and lipid content, and are typically higher for liver than for VAT. These pre-enrichment counts serve as the input reference for the cumulative recovery values reported in Table 1 and should be confirmed at Step 13 before proceeding to enrichment.

**Table 1:** Representative nuclei recovery benchmarks after centrifugation, enrichment, staining, filtration, and before BD Rhapsody loading

Sample source	n	Enrichment applied	Final nuclei concentration before loading (nuclei/μL)	Estimated total nuclei before loading	Approximate recovery from ∼1 × 10^6^ input (%)
Liver	5	Yes	1328.1 ± 207.6	8.27 × 10^5^ ± 1.29 × 10^5^	82.7 ± 12.9
VAT	6	Yes	831.2 ± 177.1	5.17 × 10^5^ ± 1.10 × 10^5^	51.7 ± 11.0

Values are shown as mean ± SD and were extracted from original BD scanner count records after centrifugation, enrichment, DyeCycle™ Green staining, and filtration, immediately before BD Rhapsody™ loading. Estimated total nuclei before loading were calculated as final nuclei concentration × final suspension volume (622.6 μL). Approximate recovery was calculated relative to the ∼1 × 10^6^ nuclei input used for enrichment. The input nuclei number was estimated by manual hemocytometer counting before enrichment and should be interpreted as an approximate starting value. These values reflect cumulative recovery during enrichment and downstream handling before loading, rather than enrichment-only recovery or formal nuclei purity quantification. Prior to enrichment, nuclei extraction yield should be verified by manual hemocytometer counting with DAPI staining (Steps 10–13); expected yields vary depending on tissue mass, quality, and lipid content, and are typically higher for liver than for VAT. NPC-derived nuclei are not subjected to magnetic enrichment and are therefore not included in this table; these samples proceed directly to DyeCycle™ Green staining (Step 27).

For nuclei directly extracted from hepatocyte-rich liver tissue or VAT, magnetic enrichment improves microscopic sample cleanliness and reduces visible debris (Figure 1). Representative BD scanner-derived benchmarks show that liver- and VAT-derived nuclei remain within a suitable concentration range for BD Rhapsody™ loading after centrifugation, enrichment, DyeCycle™ Green staining, and filtration (Table 1). Under standard conditions, starting from ∼1 × 10^6^ nuclei input, cumulative recovery is typically around 80% for liver-derived nuclei and around 50% for VAT-derived nuclei. The final nuclei concentration after enrichment and processing typically remains above 500 nuclei/μL. If the nuclei concentration falls below ∼100 nuclei/μL after filtration (Step 27e), check for potential sample loss during enrichment or centrifugation before proceeding (see troubleshooting problems 1 and 3). In contrast, NPC-derived nuclei should be processed without magnetic enrichment, as this step can result in significant nuclear loss without clear improvement in sample quality. NPC-derived nuclei are not included in Table 1 as enrichment is not applied to this fraction; these samples proceed directly to DyeCycle™ Green staining (Step 27).

For all tissue sources, nuclei should appear round to oval with intact membranes and uniform DAPI staining under bright-field microscopy. Preparations with >30% fragmented or morphologically irregular nuclei should not proceed to single-nucleus capture, as reduced library complexity and increased ambient RNA contamination are expected in such samples.

**Figure 3:**
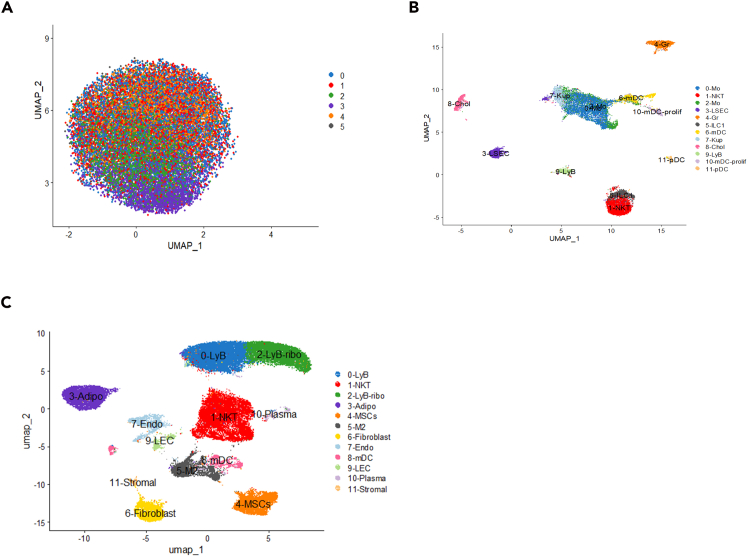
Single-nucleus RNA sequencing-based evaluation of cell-type composition UMAP visualization of shallow snRNA-seq data generated from nuclei isolated from mouse liver, NPC, and VAT samples. (A). Nucleus-level clustering of liver-derived nuclei. (B). Nucleus-level clustering of NPC-derived nuclei. (C). Nucleus-level clustering of VAT-derived nuclei.

Successful library preparation is expected to generate WTA index PCR products within the anticipated fragment-size range, as assessed by microfluidic capillary electrophoresis (Figure 2). A Qubit™ reading above 1 ng/μL and an electropherogram primary peak between ∼250 and 1000 bp are expected for all tissue sources. Shallow snRNA-seq can be used as a practical quality-control step to confirm recovery of expected major cell populations from liver, NPC, and VAT preparations before deeper sequencing or downstream biological analysis (Figure 3). When nuclei are processed using the BD Rhapsody™ System, the reported viability will read >95%, as all nuclei are expected to be DyeCycle™ Green-positive; this does not reflect actual nuclear integrity, but nuclei counts generated by the scanner remain accurate and should be used for loading calculations.

## Limitations

Anti-Nucleus MicroBeads improved the microscopic cleanliness of nuclei preparations directly extracted from hepatocyte-rich liver tissue and VAT, but the same enrichment step was not suitable for NPC-derived nuclei. In our hands, enrichment of NPC-derived samples led to marked nuclei loss, whereas liver- and VAT-derived nuclei remained compatible with downstream loading after centrifugation, enrichment, staining, and filtration (Figure 1; Table 1). Table 1 provides practical recovery benchmarks based on BD scanner counts immediately before loading. These values reflect cumulative recovery during enrichment and downstream handling, not enrichment-only recovery or formal nuclei purity quantification.

Because enrichment performance may vary across tissue types and cell populations, users should evaluate this step in small-scale pilot preparations before applying it broadly. This is particularly important for samples with smaller nuclei, distinct cellular composition, or limited starting material.

This protocol was developed using freshly collected mouse tissues. Frozen tissue may be compatible with direct nuclei extraction, but it was not formally evaluated in this workflow. Freezing may affect nuclei integrity and introduce additional transcriptomic bias. Moreover, frozen tissue is not suitable for the upstream isolation of viable NPC fractions described here. Users working with frozen samples should assess nuclei integrity microscopically and interpret downstream data with appropriate caution.

The nuclei isolation and enrichment workflow described in the sections “Extract nuclei from mouse liver, VAT, and isolated NPCs” and “Enrichment of isolated nuclei using Anti-Nucleus MicroBeads” is not intrinsically restricted to the BD Rhapsody™ HT platform and may be adapted to other single-nucleus RNA-seq systems, including droplet-based platforms (e.g., 10x Genomics Chromium) or combinatorial indexing approaches (e.g., Parse Biosciences SPLiT-seq). However, the following parameters are BD Rhapsody™ HT-specific and must be adjusted when adopting alternative systems: (i) nuclei loading concentration and volume, which vary by platform and should be determined according to the target system’s user guide; (ii) viability staining reagent, because DyeCycle™ Green is optimized for the BD Rhapsody™ scanner and should be replaced with the reagent recommended by the target platform; (iii) resuspension buffer compatibility, because Sample Buffer-RI is BD-specific and must be substituted with a compatible buffer according to the alternative platform’s guidelines; and (iv) all downstream capture, cDNA synthesis, WTA amplification, and library indexing steps, which are platform-specific and must follow the corresponding workflow.

When this protocol is applied to genetically modified or knockout mouse models, transcriptomic findings should also be interpreted in the context of strain background and residual congenic regions. Linked genomic segments surrounding targeted alleles can alter neighboring gene expression and may contribute to apparent molecular phenotypes independently of the gene of interest.^6^^,^^7^ To minimize the impact of the congenic footprint, we recommend: (i) using co-isogenic littermate controls generated from heterozygous intercrosses within a fully backcrossed (≥10 generations) line rather than separate knockout and wild-type colonies, (ii) documenting the extent of the congenic interval by SNP genotyping or RNA-seq-based haplotype mapping, (iii) systematically checking for directional expression changes ('knockout-low' pattern, referring to artifactually reduced expression of congenic-region genes in knockout versus wild-type comparisons) in genes flanking the targeted allele, which would suggest residual congenic confounding,^6^^,^^7^ and (iv) validating key transcriptomic findings at the protein level or in an independently generated line where feasible.

## Troubleshooting

### Problem 1, related to steps 1–13

When isolating nuclei from fresh mouse tissues or cells, processing delays between samples can introduce significant variability and compromise reproducibility.

### Potential solution

For experiments in which frozen-tissue processing has been validated in the user’s setting, collect tissues immediately after sacrifice and snap-freeze them in liquid nitrogen for batch nuclei isolation at a later time, ensuring uniform processing conditions. Alternatively, for experiments requiring fresh tissues or cells, process mice in parallel and collect samples simultaneously to minimize temporal gaps. To maintain sample integrity and minimize variability, process no more than four samples concurrently.

### Problem 2, related to steps 1–3

Incomplete tissue dissociation during gentleMACS™ Octo Dissociator processing using Miltenyi’s Nuclei Extraction Buffer.

### Potential solution

Limit sample input to no more than 100 mg per dissociation cycle. Mince tissue thoroughly into small pieces (1–2 mm fragments) before loading into the gentleMACS™ Octo Dissociator. Include an additional 5–10 min on-ice incubation after the dissociation program to enhance tissue lysis and improve nuclei release.

### Problem 3, related to steps 4 and 8

A substantial loss of nuclei occurs after filtering the nuclei suspension through MACS SmartStrainer (70 μm in Step 4; 30 μm in Step 8).

### Potential solution

Ensure SmartStrainers are thoroughly pre-washed before use to minimize nuclei retention on the membrane when significant nuclei loss is observed. For nuclei isolated from other tissue types, first follow the filtration sequence described in this protocol. If significant nuclei losses persist, substitute a MACS SmartStrainer (100 μm) instead of the 30 μm, then proceed directly to nuclei enrichment or to Single-nucleus capture and cDNA Synthesis, skipping Step 8.

### Problem 4, related to steps 35, 36, 40, and 44

Low cDNA yield or low library yield is observed after reverse transcription, amplification, or indexing PCR.

### Potential solution

Low cDNA or library yield may result from low nuclei input, nuclei loss during processing, incomplete reverse transcription, insufficient mixing, suboptimal incubation, or an inappropriate PCR cycle number. Verify nuclei count and integrity before library preparation and ensure that the nuclei suspension is homogeneous before loading. During reverse transcription, mix gently but thoroughly and maintain the recommended incubation conditions. Adjust the PCR cycle number according to nuclei input as described in the protocol. Avoid delays between nuclei preparation, capture, reverse transcription, and downstream amplification.

### Problem 5, related to steps 1–45

Low gene detection per nucleus or reduced transcript complexity is observed after sequencing.

### Potential solution

This problem is commonly associated with RNA degradation, poor nuclei integrity, over-lysis, prolonged processing, low nuclei input, or nuclei loss during filtration or enrichment. Minimize the time between tissue collection, nuclei isolation, and capture, and keep samples on ice unless otherwise specified. Check nuclei integrity microscopically before loading and avoid over-lysis during extraction. For NPC-derived nuclei, skip magnetic enrichment because this step can markedly reduce recovery without improving sample quality. If low gene detection persists, verify nuclei concentration, input number, library quality, sequencing depth, and whether the sample type has intrinsically low RNA content.

### Problem 6, related to steps 1–8

Lower nuclei quality or yield when processing previously frozen tissue compared with fresh material.

### Potential solution

If fresh material is unavailable, snap-freeze tissue pieces of ≤50 mg in liquid nitrogen immediately after dissection and store at −80 °C. When processing frozen material, avoid repeated freeze-thaw cycles and transfer tissue directly into pre-chilled nuclei extraction buffer without prior thawing. Assess nuclei integrity by DAPI staining and bright-field microscopy (Steps 10–13) before proceeding; preparations with >30% fragmented nuclei or extensive debris may yield libraries of reduced complexity and increased ambient RNA contamination. Note that frozen tissue is not compatible with the upstream viable NPC isolation step, which requires fresh tissue.

## Resource availability

### Lead contact

Further information and requests for resources and reagents should be directed to and will be fulfilled by the lead contacts, Cosmin Sebastian Voican (cosmin.voican@aphp.fr).

### Technical contact

Technical questions on executing this protocol should be directed to and will be answered by the technical contact, Lei Li (lei.li@universite-paris-saclay.fr).

### Materials availability

This study did not generate new unique reagents.

### Data and code availability

Original BD scanner count records supporting Table 1, representative microscopy images supporting Figure 1, electropherogram files supporting Figure 2, and processed shallow snRNA-seq quality-control data supporting Figure 3 are available from the lead contact upon reasonable request. Raw shallow sequencing data have not been deposited in a public repository because they were generated for protocol quality-control purposes only and are not used for biological inference in this study. This protocol did not generate original code.

## Acknowledgments

C.S.V. was supported by 10.13039/501100001677INSERM, 10.13039/501100007241Université Paris-Saclay, AP-HP, and ANR (ANR-23-CE18-0035-03). L.L. received a PhD scholarship from the Chinese Scholarship Council (CSC). J.G.P. was supported by the 10.13039/501100003323Agence nationale de recherches sur le sida et les hépatites virales | Maladies infectieuses émergentes (ANRS MIE), SIRIC Cancer research and personalized medicine (CARPEM), Domaine de recherche et d’innovation majeur-Immunothérapies, auto-immunité et cancer (DIM-ITAC, Région Île-de-France), and 10.13039/501100006364Institut national du cancer (INCa PLBIO21-212, INCa_18542). Animal experiments were supported by the ANIMEX platform, affiliated with the Paris-Saclay Institute for Innovative Therapeutics (IPSIT-SFR/US31-UMS3679). Sequencing was supported by the BIOMICS platform (10.13039/501100003762Pasteur Institute, Paris, France). We thank Laurence Monnet (BD Biosciences) for technical guidance and experimental support.

## Author contributions

L.L. optimized the protocols, performed data collection and analysis, and wrote the initial draft of the manuscript. J.G.P. and C.S.V. collected data related to nuclei enrichment, single-nucleus capture, and cDNA synthesis. C.D. performed data analysis of shallow sequencing. W.H., N.T., and M.B. assisted with animal experiments. C.S.V., J.G.P., M.L.G., M.C.M., G.K., G.P., and A.-M.C. provided scientific guidance and critically evaluated the protocols, study design, and manuscript.

## Declaration of interests

J.G.P. is the inventor of patents covering the diagnosis, prognosis, and treatment of cancers, including patents licensed to Turnstone Biologics and Therafast Bio. G.K. has been holding research contracts with Daiichi Sankyo, Eleor, Kaleido, Lytix Pharma, PharmaMar, Osasuna Therapeutics, Samsara Therapeutics, Sanofi, Sutro, Tollys, and Vascage. G.K. is on the Board of Directors of the Bristol Myers Squibb Foundation France and on the Scientific Advisory Board of Servier Institute. G.K. is a scientific co-founder of everImmune, Osasuna Therapeutics, Samsara Therapeutics and Therafast Bio. G.K. is the inventor of patents covering therapeutic targeting of aging, cancer, cystic fibrosis, and metabolic disorders. G.K.’s brother, Romano Kroemer, was an employee of Sanofi and now consults for Boehringer-Ingelheim. G.K.’s wife, Laurence Zitvogel, has held research contracts with Glaxo Smyth Kline, Incyte, Lytix, Kaleido, Innovate Pharma, Daiichi Sankyo, Pilege, Merus, Transgene, 9 m, Tusk and Roche, was on the Board of Directors of Transgene, is a cofounder of everImmune, and holds patents covering the treatment of cancer and the therapeutic manipulation of the microbiota. The funders had no role in the design of the study, in the writing of the manuscript, or in the decision to publish the results.

## Declaration of generative AI and AI-assisted technologies in the writing process

During the preparation of this work, the authors used ChatGPT (OpenAI) to assist with language editing and text polishing. After using this tool, the authors reviewed and edited the content as needed and take full responsibility for the content of the published article.
